# Emotional valence and spatial congruency differentially modulate crossmodal processing: an fMRI study

**DOI:** 10.3389/fnhum.2014.00659

**Published:** 2014-08-27

**Authors:** Dhana Wolf, Lisa Schock, Saurabh Bhavsar, Liliana R. Demenescu, Walter Sturm, Klaus Mathiak

**Affiliations:** ^1^Department of Psychiatry, Psychotherapy and Psychosomatics, Medical School, RWTH Aachen UniversityAachen, Germany; ^2^Interdisciplinary Centre for Clinical Research, Medical School, RWTH Aachen UniversityAachen, Germany; ^3^JARA–Translational Brain Medicine, Research Centre Jülich, JülichAachen, Germany; ^4^Department of Neurology, Clinical Neuropsychology, Medical School, RWTH Aachen UniversityAachen, Germany

**Keywords:** hemodynamic mismatch response, spatial congruency, crossmodal spatial cueing, attention, valence, auditory cortex

## Abstract

Salient exogenous stimuli modulate attentional processes and lead to attention shifts–even across modalities and at a pre-attentive level. Stimulus properties such as hemispheric laterality and emotional valence influence processing, but their specific interaction in audio-visual attention paradigms remains ambiguous. We conducted an fMRI experiment to investigate the interaction of supramodal spatial congruency, emotional salience, and stimulus presentation side on neural processes of attention modulation. Emotionally neutral auditory deviants were presented in a dichotic listening oddball design. Simultaneously, visual target stimuli (schematic faces) were presented, which displayed either a negative or a positive emotion. These targets were presented in the left or in the right visual field and were either spatially congruent (valid) or incongruent (invalid) with the concurrent deviant auditory stimuli. According to our expectation we observed that deviant stimuli serve as attention-directing cues for visual target stimuli. Region-of-interest (ROI) analyses suggested differential effects of stimulus valence and spatial presentation on the hemodynamic response in bilateral auditory cortices. These results underline the importance of valence and presentation side for attention guidance by deviant sound events and may hint at a hemispheric specialization for valence and attention processing.

## Introduction

For an efficient interaction with the environment, information from various sensory modalities is integrated into a unified spatial representation. Since the processing capacity of perceptual input is limited, spatial attention needs to be selectively allocated to relevant stimuli. The importance of such a mechanism is reflected, for instance, in the huge impact of impairments of attention in the left visual field—unilateral neglect—on simple activities of daily living in patients with right-hemisphere damage (for a review see Danckert and Ferber, 2006). The allocation of attention is guided by the processing and evaluation of information from various sensory modalities as well as top-down control mechanisms. The underlying neural mechanisms of attention allocation are modulated by different stimulus properties such as presentation side and emotional content.

### Spatial cueing and spatial congruency

Across modalities, attention networks interact at a supramodal level to guide attention to the most important stimulus in the environment. Attention distribution mechanisms modulate processing in the target-modality sensory cortices and also in other, usually to be ignored modalities. This was demonstrated for audio-visual, tactile-visual, and tactile-auditory interactions (for a review, see Eimer and Driver, 2001). Thus, attention shifts following exogenous cues influence target detection even across modalities (Eimer and Driver, 2001; McDonald et al., 2003; Menning et al., 2005).

When a sensory stimulation is presented on the same side as the target stimulus, this valid cue reduces reaction time. When the stimulation is presented on the opposite side (invalid cue), attention is directed away from the target location, leading to increased reaction time (Posner, 1980). The auditory oddball paradigm is a powerful mean to investigate this cueing effect at a pre-attentive level. The paradigm comprises deviant auditory stimuli presented in a rapid sequence of frequent standard stimuli. Deviants of any type implemented in a series of standard events constitute a violation of the established pattern and elicit a mismatch response in the auditory cortex, triggering attentional shifts to the side of the deviant (Schröger, 1996; Schock et al., 2013). Schröger et al. combined an auditory oddball design presented on a to-be-ignored ear with an auditory GoNogo-task on the other ear. They found a prolonged reaction time to the task when it was preceded by a deviant sound on the ignored ear. This was interpreted as a shift of attention induced by the deviant sound.

This oddball-induced mismatch response is a well-established tool in the investigation of neural responses (EEG: Näätänen et al., 1989; Schröger, 1996; Näätänen et al., 2004; MEG: Mathiak et al., 2005; fMRI: Mathiak et al., 2002; Schock et al., 2012). These studies reported that even non-attended changes in the auditory stream induce increased activation of the auditory cortex. Further, this effect is modulated by attention (Alho et al., 1999; Zvyagintsev et al., 2011), cross-modal information (Calvert and Campbell, 2003; Kayser et al., 2005, 2007; Zvyagintsev et al., 2009) and top-down regulation (van Atteveldt et al., 2007; Zvyagintsev et al., 2009; Hsieh et al., 2012).

### The influence of valence on attention distribution

Emotional valence, an important factor contributing to the salience of stimuli, influences attention-related processing. Negative valence draws attention effectively and enhances stimulus processing (Eastwood et al., 2001; Fenske and Eastwood, 2003; Rowe et al., 2007). A negative stimulus can narrow the attentional focus accompanied by enhanced processing. In visual search, negative stimuli are found faster and with fewer errors than positive stimuli (Eastwood et al., 2001). In a preceding behavioral study, Schock et al. (2013) combined schematic faces—representing emotionally salient target stimuli—with dichotic syllables presented in an oddball paradigm. Visual and auditory stimuli were presented on the same side (spatially congruent) or on opposite sides (spatially incongruent). Prolonged response time was found to left-lateralized spatially incongruent stimuli if they were of positive valence, while emotionally negative targets were not affected by spatial incongruency. This behavioral effect may be induced by pre-attentive auditory processing.

### Aims and hypotheses

Although neural mechanisms of attention have been extensively studied, the influence of emotional valence, hemispheric lateralization, and their specific interactions on early auditory processing, remain ambiguous. The goal of the present study was to investigate the interaction of emotional valence, visual field, and spatial congruency on auditory cortex activation. For this aim, we conducted an fMRI experiment combining a dichotic listening oddball design with emotional visual targets. We hypothesized that deviant sounds would trigger a shift of attention towards the side of the deviant and thereby accelerate response time to spatially congruent visual stimuli, while response time to spatially incongruent stimuli would be increased. Emotional salience was expected to interact with spatial congruency, in that negative salience would reduce this reaction time costs in spatially incongruent trials. Regarding the neurophysiological data, we hypothesized that auditory cortex responses to deviant sounds would be modulated by spatial congruency with, and emotion of, the target faces. Furthermore, attention and salience-related networks were expected to respond stronger to spatially incongruent than to congruent stimuli.

## Materials and methods

We conducted an fMRI experiment combining a dichotic listening oddball design with emotional visual targets. A deviant stimulus would induce an attention shift to the side of the deviant. Visual target stimuli were schematic faces that exclusively displayed the feature of positive and negative face expressions. They were presented in the left or right visual field either spatially congruent (valid) or incongruent (invalid) with the auditory deviants. Participants were instructed to ignore the auditory modality and to indicate the detection of a visual target stimulus via button press. We have successfully applied this paradigm in a previous behavioral study and demonstrated significant interactions of valence and visual field and of valence and spatial congruency on reaction time (Schock et al., 2013).

### Subjects

Sixteen healthy volunteers (8 females, age 21–36 years, mean age 24.9 ± 4.5 years) participated in the study. Fifteen subjects were right-handed, as indicated by the laterality quotient of the Edinburgh Inventory (Oldfield, 1971) and one subject was ambidextrous. Subjects were screened with the Structured Clinical Interview (SCID-I; Wittchen et al., 1997) for the diagnostic and Statistical Manual of Mental Disorders (DSM-IV) to exclude subjects with psychiatric disorders. The study was approved by the local Ethics Committee of the Medical School of the RWTH Aachen University and was conducted in accordance with the Code of Ethics of the World Medical Association (Declaration of Helsinki). Informed consent was obtained from all subjects.

### Stimuli

#### Auditory stimuli

The stimuli comprised two consonant-vowel syllables (/ba/ and /ka/) presented in a dichotic listening oddball design. In 90% of the presentations the standard /ba/ was presented to both ears. In the remaining 10% the deviant /ka/ was presented to one ear (5% to the left ear and 5% to the right ear) and the standard /ba/ to the other ear (left/ba/-right/ka/ and left/ka/-right/ba/). The syllables were delivered synchronous over both ears, with equal sound level and duration (300 ms), and with a constant stimulus onset asynchrony (SOA) of 500 ms.

#### Visual stimuli

Visual stimuli were composed of schematic drawings of faces with either a positive or negative emotional expression. The positive expression included a half-elliptic mouth shape and round-shaped eyebrows. The negative expression was achieved by inverting the mouth downwards and orienting the inner eyebrow ends upwards. These expressions were validated beforehand and were rated as either happy or sad (see Schock et al., 2013). For trials including a face, the visual stimuli were presented 175 ms after auditory stimulus onset and lasted for 150 ms.

### Design

**Figure 1:**
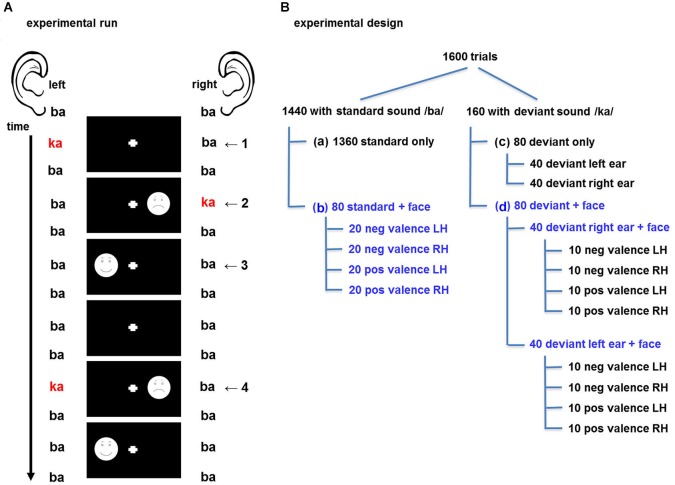
**(A)** Design of the experimental run. A dichotic listening oddball paradigm was presented to elicit crossmodal cueing effects for the visuospatial stimuli. Auditory stimuli comprised the consonant-vowel syllables binaural /ba/ as standards and dichotic /ka/-/ba/, resp. /ba/-/ka/, as deviants. Schematic drawings of faces with positive and negative valence probed emotional salience effects. In the audio-only condition, the syllables and the fixation cross were presented (1). For spatially congruent (valid) stimuli, the deviant and the face were presented in the same hemifield (2); in the incongruent (invalid) condition, stimuli were in opposite hemifields (4). Further half of the faces were without preceding deviant (3). Standard syllables with fixation cross only constituted the baseline condition. **(B)** Experimental design with balanced factors: One 14-min session comprised 1600 trials; 10 percent of which comprised a deviant stimulus. The design accounted for a balanced distribution of deviant auditory stimuli and visual stimuli throughout the sequence; with an equal distribution of the factors spatial congruency, valence, and presentation side (blue writing: balanced factors). Altogether, 14 trial types were defined in 1 baseline and 3 event types: **(a)** “standard only” (baseline), **(b)** “standard + face”, **(c)** “deviant only”, **(d)** “deviant + face”. pos = positive valence “happy”, neg =negative valence “sad”, LH = face presented in left hemifield, RH = face present in right hemifield.

The experiment comprised three sessions of 14 min each. In each session, 1600 trials were presented in a rapid, event-related design. A trial was defined as the presentation of one auditory stimulus with or without a face stimulus, so that the length of each trial corresponded to the auditory stimuli onset asynchrony of 500 ms (Figure 1A). Audio-visual trial types were specified by the factors *valence* (positive vs. negative facial expression), *visual field* (left vs. right visual field), and *ear* (left- vs. right-ear deviant). In total 14 events of interest emerged: 4 times face and standard sound (face left/right, positive/negative), 2 distinct deviants (left/right), and 8 events comprising a face and a deviant (combination of the previous factors). These trial types are displayed in Figure 1B. Each session comprised the baseline condition with 1440 trials with “standard only” event types (standard auditory event without visual stimulus; Figure 1B-a), 80 trials with the four “standard + face” event types (20 each, Figure 1B-b), 80 trials with two “deviant only” event types (40 each, Figure 1B-c) and 80 trials with eight “deviant + face” event types (10 each, Figure 1B-d). In 50% of the cases the auditory and visual stimulus were presented on the same side (spatially congruent, “valid”) and in the other 50% the stimuli were presented on the opposite side (spatially incongruent, “invalid”; see Figure 1A).

The design accounted for a balanced distribution of auditory deviants and visual stimuli throughout the oddball sequence. A minimum of two standards (left/ba/-right/ba/) were placed between two deviants (left/ba/-right/ka/ or left/ka/-right/ba/). Likewise, at least two trials without visual stimulation were presented between two trials with face presentation to prevent a button press overlap. Each session was individually randomized: first the deviant distribution was randomized. Then, left-ear and right-ear deviant events were randomly selected and assigned to the “deviant + face” events and in a third step the standard events were randomly selected and assigned to the “standard + face” events.

Participants were instructed to ignore the sound and report detection of the face stimulus via button pressing. Visual stimuli were displayed via MR-compatible video goggles (Visua-StimDigital, Resonance Technology, RT, Northridge, CA, USA) and sounds were delivered with MR-compatible headphones. Sound levels were adjusted to comfortable hearing level and good audibility during scanning. Stimulus presentation and response time recording was performed using the software *Presentation* (Version 10.0; Neurobehavioral Systems, Inc., Albany, CA).

### MR imaging

Functional imaging was conducted on a 3T Magnetom Trio MR scanner (Siemens Medical Systems, Erlangen, Germany) in the department of Psychiatry, Psychotherapy and Psychosomatics at the Medical School of RWTH Aachen University. Functional images were collected with echo planar imaging (EPI) sensitive to blood oxygenation level dependent (BOLD) contrast (interleaved acquisition of 34 slices; repetition time [TR] = 2000 ms; echo time [TE] = 28 ms; flip angle [FA] = 77°; slice thickness = 3 mm; gap 0.75 mm; matrix size = 64 × 64; field of view [FOV] = 192 × 192 mm^2^; voxel size = 3 × 3 × 3 mm^3^). Slices covered the entire cerebral cortex and were positioned oblique-transversally to achieve maximal brain coverage. 420 volumes were collected per session, of which the first seven were discarded to remove the influence of T1 saturation effects. Head movement was minimized with the use of foam wedges to securely hold the head in the 12-channel head coil. Structural images were obtained using a high-resolution T1-weighted 3D-sequence (TR = 1900 ms; inversion time TI = 900 ms; TE = 2.52 ms; FA = 9°; FOV = 256 × 256 mm^2^; 176 3D-partitions with an isotropic resolution of 1 mm^2^).

### Behavioral data analysis

Button presses in response to visual stimuli were analyzed by subtracting the onset of the visual stimulus from the onset of button press. The mean reaction time (in ms) was calculated for each event type and each participant. A 2 × 2 × 2 repeated-measures ANOVA with the factors *valence* (positive, negative emotion), *visual field* (left, right), and *spatial congruency* (auditory and visual presentation on the same/opposite side) was conducted. Significance level was set at *p* < 0.05 after Bonferroni correction. Paired *t*-tests disentangled the effects *post-hoc*.

### Imaging data analysis

Functional MRI data analysis was conducted with the Statistical Parametric Mapping software (SPM8^1^; implemented in MATLAB) (MathWorks, Natick, MA, USA). After discarding the first seven volumes, 413 volumes per session from each participant were spatially realigned to the mean image to correct for head movement, normalized to the stereotaxic anatomical MNI (Montreal Neurological Institute) space with 2 mm isotropic voxels, and spatially smoothed with an 8 mm (FWHM) isotropic Gaussian kernel to account for inter-subject variability in brain anatomy and to increase signal-to-noise ratio. A rapid event-related design was chosen to model the experimental conditions (14 audio-visual events, see Section Design) with a general linear model (GLM).

#### Whole brain analysis

The contrast “deviant without face” vs. “standard without face” (baseline) was built to document auditory cortex activation in response to deviant stimuli with a *t*-test. Results were corrected with a family-wise-error (FWE) rate of *p* < 0.05 to account for multiple testing.

To investigate the neural mechanisms underlying spatial congruency effects, a *t*-contrast was designed, which compared spatially incongruent trials (“left deviant + right face”, and “right face + left deviant”) with spatially congruent trials (“left deviant + left face”, and “right deviant + right face”).

#### Region-of-interest (ROI) analysis

**Figure 2:**
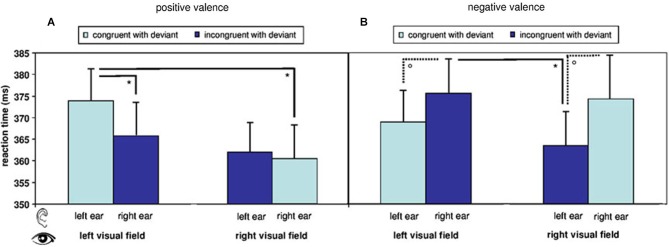
**Reaction times in response to visual target stimuli during fMRI**. Reaction times are modulated by a main effect of stimulus valence and by an interaction of valence, visual field of presentation, and spatial congruency of visual with auditory deviant. Overall reaction time pattern reflects a difference in processing of positive and negative stimuli with regard to presentation side. Postdoc *T*-tests further illustrate the findings: **(A)** Reaction times to visual stimuli in the right visual field were faster than to those in the left visual field in the positive congruent condition and **(B)** in the negative incongruent conditions. (°: *p* < 0.1; *: *p* (uncorr.) < 0.05; mean ± SE).

The contrast estimates for each subject were extracted from bilateral auditory activation cluster peak voxel of the contrast “deviant without face” vs. “standard without face” (baseline; Figure 2, Table 1). The response amplitudes at bilateral Auditory cortex (AC) were assessed by a 2 × 2 × 2 repeated-measures ANOVA, with the factors stimulus *valence* (positive, negative), *visual field* (left, right) and *ear* of deviant presentation (left, right). For this analysis, only trials comprising a deviant and a face were considered.

**Table 1:** **Deviance responses**.

Contrast	Anatomical area	Hemisphere	BA	MNI coordinates			Peak *T*-values	Cluster size [voxel]
				*X*	*Y*	*Z*
**“deviant only” vs. “standard only”**	Superior temporal gyrus	R	42	68	−26	8	6.80	229
	Superior temporal gyrus	L	22	−62	−24	4	5.77	56

*Threshold p < 0.05, FWE-corr. and cluster extent ≥ 50 voxels, BA: Brodmann Area, MNI: Montreal Neurological Institute*.

## Results

### Behavioral results

The here applied, uninformative cueing paradigm yielded no speeding of reaction times after auditory deviants (368.10 ± 28.98 ms) as compared to standards (367.13 ± 25.39 ms; *T*_15_ = 0.575, *p* = 0.574, n.s.). A repeated-measure ANOVA including the factors *spatial congruency*, *visual field*, and *valence* of the visual stimulus analyzed the reaction times in response to stimuli after deviants. A significant main effect emerged for *valence* (*F*_[1,15]_ = 7.820, *p* = 0.014). Mean differences (±SE) yielded significantly faster reactions to positive as compared to negative targets (−5.010 ± 1.792). The factor *spatial congruency* yielded a trend-level effect (*F*_[1,15]_ = 3.476, *p* = 0.082), while *visual field* did not yield significant effects (*F*_[1,15]_ = 2.305, *p* = 0.150, n.s.). However, a significant result emerged for the triple interaction between *visual field*, *valence*, and *spatial* *congruency* (*F*_[1,15]_ = 9.262, *p* = 0.008). In the *post-hoc* *t*-tests with Bonferroni-correction for multiple comparisons, no contrast between the eight audio-visual conditions yielded significance (see descriptive analysis in Figure 2).

### fMRI results

#### Whole brain analysis

**Figure 3:**
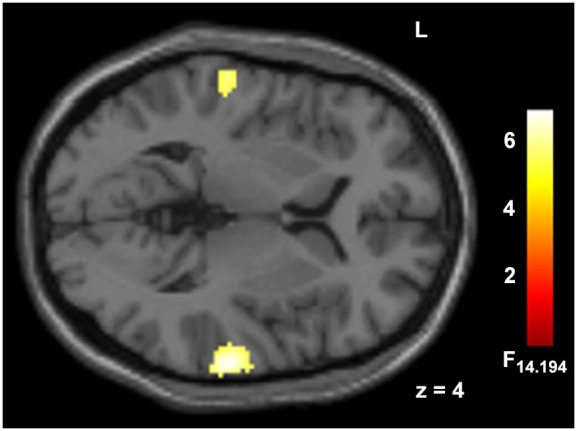
**Hemodynamic responses to auditory deviants.** Deviant syllables (/ka/) as compared to ongoing standards (/ba/) elicited responses in auditory cortex. Peak voxel MNI coordinates of right AC: [68, −26, 8] and left AC: [−62, −23, 4]; *p* < 0.05, FWE corr., extend threshold 5 voxels.

Deviant auditory stimuli yielded large cluster in bilateral AC (*p* < 0.05, FWE corr., Figure 3, Table 1). The incongruency contrast, comparing spatially incongruent with congruent trials, did not reveal significant effects in a whole-brain contrast.

#### ROI analysis

**Figure 4:**
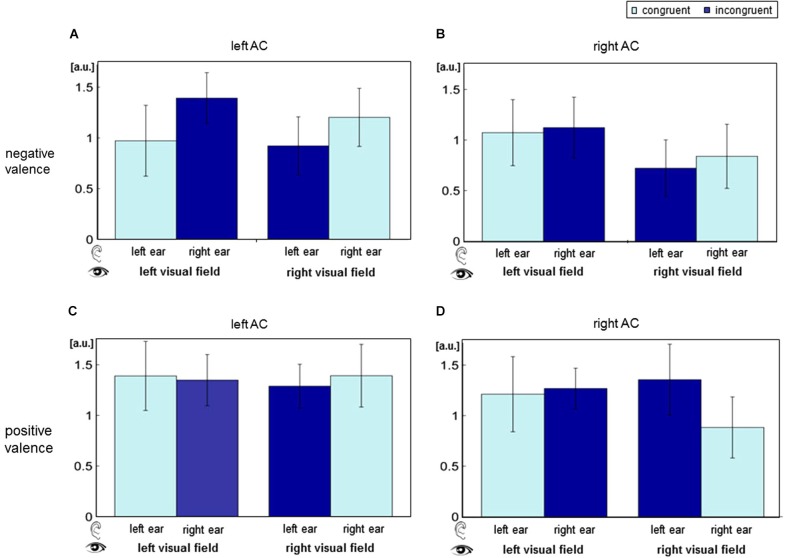
**Auditory cortex (AC) responses for the distinct stimulus combinations of the factors *ear, visual field* and *valence***. BOLD responses were extracted for all audio-visual deviants separately at the left (panels **A** and **C**) and the right AC (**B** and **D**). In the upper row (**A** and **B**), visual stimuli had negative valence; in the lower row (**C** and **D**), valence was positive. ANOVA revealed significant main effect of visual field and an interaction of valence and visual field. a.u.: arbitrary units; AC: auditory cortex.

The contrast estimates of the contrast “deviant” vs. “standard” were extracted from the cluster peak voxel in bilateral AC (MNI coordinates [68, −26, 8] and [−62, −23, 4]; Figure 4). *Visual field* yielded a significant main effect (left AC: *F*_[1,15]_ = 6.64, *p* = 0.0112; right AC: *F*_[1,15]_ = 10.04, *p* = 0.0019), and *ear* (deviant presentation side) yielded a main effect in the right AC (*F*_[1,15]_ = 9.94, *p* = 0.002). Valence trended toward significance in the right AC (*F*_[1,15]_ = 3.16, *p* = 0.078). Furthermore, a significant interaction of *visual field* and *valence* emerged in both ACs (left AC: *F*_[1,15]_ = 4.29, *p* = 0.0405; right AC: *F*_[1,15]_ = 16.4, *p* < 0.001). Spatial congruency (interaction of *ear* and *visual field*) failed a significant effect (left AC: *F*_[1,15]_ = 0.01, *p* = 0.918, n.s.; right AC: *F*_[1,15]_ = 0.13, *p* = 0.717, n.s.). Likewise, significance failed for the interaction of *ear* and *valence* (left AC: *F*_[1,15]_ = 0.57, *p* = 0.451; right AC: *F*_[1,15]_ = 0.82, *p* = 0.367), as well as a triple interaction of *ear*, *visual field*, and *valence* (left AC: *F*_[1,15]_ = 0.01, *p* = 0.908; right AC: *F*_[1,15]_ = 0.45, *p* = 0.503).

## Discussion

In the present study neural correlates of attention processes in a dichotic listening oddball paradigm were investigated. An auditory stimulus (standard or deviant) preceded visual target presentation (positive and negative schematic faces in the left or right visual field). Events comprising a deviant auditory stimulus were equally distributed between spatially congruent and incongruent trials, thus constituting uninformative spatial cues with a 50% validity ratio. In analogy to the cueing effect of Posner (1980), but applying uninformative crossmodal cues (Spence and Driver, 1997; Ward et al., 2000; Mazza et al., 2007), the auditory deviants were expected to serve as crossmodal spatial cues for visual target stimuli. Indeed, presentation side and valence of visual stimuli significantly affected AC response.

### Behavioral data

To our knowledge, this is the first study to combine schematic faces as emotional stimuli with a spatial cueing (mismatch auditory oddball) design in fMRI. The effectiveness of schematic faces for realistic emotion display (Dyck et al., 2008) and for affective facial stimuli in modulating the allocation of attention in behavioral studies has been demonstrated previously. Fox et al. (2001) used schematic faces as spatially incongruent cues in a visual paradigm and demonstrated that negative faces draw and hold attention effectively. In a similar vein, other studies have demonstrated comparable valence effects for the presentation of schematic, or cartoon faces (Eastwood et al., 2001; Fenske and Eastwood, 2003; Santos et al., 2011).

In our previous behavioral study employing the here reported paradigm, we observed that reaction time in response to a visual stimulus following a deviant auditory stimulus was facilitated. Furthermore, reaction time costs emerged in response to spatially incongruent stimuli only in the positive valence condition, whereas negative valence overcame this incongruency effect (Schock et al., 2013). The interaction between spatial presentation and emotional valence of target stimuli is a well-documented phenomenon (Eastwood et al., 2001; Fenske and Eastwood, 2003). The current findings partially reflect the previously observed effects (Schock et al., 2013). Deviant sounds preceding a face did not reduce reaction times compared to faces preceded by a standard sound, but a main effect of *valence* and an interaction of spatial* congruency*, *visual field* and *valence* was replicated. In summary, alerting may not have been efficient in the noisy MR-environment, but the overall reaction time pattern reflects a difference in processing of positive and negative stimuli with spatial congruency constituting a modulating factor.

### Effect of deviants and spatial incongruency

Contrasting the presentation of a deviant sound with a standard, deviance yielded increased hemodynamic responses in bilateral AC. Increased AC activation in response to non-attended deviants within an oddball listening design is well established (EEG: Sams et al., 1985; Näätänen et al., 1989, 2004; MEG: Alho et al., 1998; Mathiak et al., 2000; Phillips et al., 2000; fMRI: Mathiak et al., 2002; Schock et al., 2012). This effect on very early processing steps may underlie the redirection of attention toward changing features in the environment (Näätänen, 1995). Deviant stimuli were presented either spatially congruent or incongruent with a visual target. Contrasting our hypothesis, no spatial congruency effect emerged at the whole-brain level. Our design, comprising uninformative cues (equal probability of presentation with or without a face) and a wide range of stimulus combinations (valence, visual field, and spatial congruency) may have been inadequate for this question. Until now, only few fMRI studies have investigated whole-brain activation changes in response to spatially incongruent vs. congruent audiovisual stimuli. Sestieri et al. (2006) reported increased response of superior temporal sulcus to spatially congruent audiovisual stimuli, as compared to spatially incongruent stimuli. However, in contrast to our paradigm, participants were explicitly asked to pay attention to both the visual and the auditory stimuli.

### Effects of deviance and visual field on AC processing

ANOVA analyses of AC peak voxel responses revealed a main effect of *visual field* and an interaction effect of *visual field* and *valence* in both AC. Furthermore, a trend-level effect of *valence* emerged in the right AC.

Up until now, the direct comparison of AC responses to spatially incongruent vs. congruent audiovisual stimuli has not been investigated with an auditory oddball design and fMRI. In an EEG study, Teder-Sälejärvi et al. (2005) compared the ERP of bimodal audiovisual stimuli in spatially congruent and incongruent conditions. For incongruent stimulus pairings, the authors reported a shift in phase and amplitude of activity at 100–400 ms, which were allocated to ventral occipito-temporal cortex. Spatially congruent pairings yielded an amplitude modulation of activity at 260–280 ms, localized to superior temporal regions. Though this result hints at an effect of spatial congruency on early auditory processing, we did not find an interaction of *ear* of presentation and *visual field* (i.e., spatial congruency). However, the significant effect of deviance and of visual field of face presentation on AC response suggests a similar notion.

In line with our results, several studies reported that visual input can modulate auditory processing (Kayser et al., 2007; Zvyagintsev et al., 2009; Hsieh et al., 2012). Direct connections between primary auditory and visual cortex have additionally been identified by anatomic studies in primates and fMRI connectivity analysis (Eckert et al., 2008).

### Effects of emotional valence on AC processing

The influence of emotion and valence of visual stimuli on early auditory processing has been reported in a wide range of electrophysiological studies. In an EEG-experiment (Alexandrov et al., 2007) emotional context (monetary reward or punishment) led to significantly larger auditory cortex event-related potentials in response to negative as compared to positive trials. Visually induced emotional states with positive or negative pictures have also been reported to modulate event-related potentials of auditory stimuli (Surakka et al., 1998; Yamashita et al., 2005; Sugimoto et al., 2007; Domínguez-Borràs et al., 2009; Wang et al., 2009). Functional MRI studies complement this picture: Schock et al. (2012) observed right lateralized prefrontal cortex (PFC) activation and enhanced processing of right-ear deviants in the sad mood condition. Fear conditioning, as conveyed by visual stimuli, also affected AC activity (Armony and Dolan, 2001). AC response to fear-conditioned stimuli was modulated by the presence of a visual context for the likelihood of aversive stimuli appearance.

Complementing these studies, we were the first to investigate face valence effect on AC response in the context of spatial congruency (induced by deviating syllables in an auditory oddball design) and visual field of presentation. The significant interaction of valence and visual field indicates that emotional valence of visual stimuli may modulate auditory processing at an early stage. This finding may support the hypotheses of hemispheric specialization for multimodal integration and emotion processing. According to the valence hypothesis, emotional contexts, especially negative stimuli, are primarily lateralized to the right hemisphere, while positive stimuli are dominantly processed in the left hemisphere (for a review of the valence hypothesis, see Demaree et al., 2005). Similarly, Schock et al. reported enhanced ipsilateral processing of right-ear deviants in the right AC during sad mood, but not during happy or neutral mood. Further, Petit et al. report a right hemispheric enhancement in frontal and temporal areas in response to unattended deviant tones (Petit et al., 2007). Although recent meta-analyses of neuroimaging studies investigating laterality effects on emotional face processing were not able to confirm the valence hypothesis (Wager et al., 2003; Fusar-Poli et al., 2009), our results suggest that within multimodal settings, processing of deviant tones may be affected by laterality and valence—at least at an early auditory stage.

### Limitations

To our knowledge, this study is the first to combine fMRI measurement with a dichotic listening oddball design and emotional faces as target stimuli. The resulting high number of conditions and parameters are challenging to take into account. Due to limitations in paradigm length, visual stimuli with neutral emotion were not implemented. Furthermore, deviant oddball paradigms yield comparably small number of events for subsequent analyses since a subdivision into the large number of varying conditions is necessary. However, increasing the experiment duration may induce unwanted effects on attention and arousal. This design-intrinsic small event number as well as a relatively low number of participants may account for the missing results of the whole brain contrasts. The neural response to deviants was clearly detectable in the AC, yet this observation was not fully reflected in the behavioral responses. Our inability to fully reproduce the behavioral effect of spatially congruent stimuli may be explained by the fMRI measurement. The difficulty to reproduce behavioral data within the scanner environment and concurrent reaction time slowing is frequently reported (Plank et al., 2012). Participants in our fMRI experiment responded on average 40 ms slower and with a larger deviation than participants in the behavioral study utilizing the same paradigm (Schock et al., 2013). The fMRI environment may create increased arousal and ongoing distraction due to scanner noise. Indeed, Skouras et al. (2013) recently reported an effect of scanner noise on affective brain processes. Therefore, the fMRI environment may have prevented any salience-driven response acceleration in our study. Nevertheless, although the visual target stimuli exhibit clear valence, the stimuli’s arousal value is rather low. Future studies may address the question of stimulus arousal and valence differences. Higher arousal values may increase the observed effects of valence and visual presentation side. Furthermore, a neutral condition could be implemented to further disentangle emotion and attention effects and laterality of hemispheric activation.

## Conclusion

Deviant stimuli of a dichotic listening oddball design were shown to serve as attention-directing cues for visual target stimuli during fMRI measurement. Deviant events and visual targets of different valence induced supramodal modulation of auditory cortices. This modulation was significantly dependent on visual field of presentation and on the interaction of valence and visual field. The present results underline the importance of valence and presentation side for attention guidance by deviant sound events and support the notion of intermodal effects on auditory processing, which may be modulated by emotions.

## Conflict of interest statement

The authors declare that the research was conducted in the absence of any commercial or financial relationships that could be construed as a potential conflict of interest.

## Footnotes

^1^www.fil.ion.ucl.ac.uk
